# Utility of the Anterior Oblique-Viewing Endoscope and the Double-Balloon Enteroscope for Endoscopic Retrograde Cholangiopancreatography in Patients with Billroth II Gastrectomy

**DOI:** 10.1155/2012/389269

**Published:** 2012-09-30

**Authors:** Manabu Sen-yo, Seiji Kaino, Shigeyuki Suenaga, Toshiyuki Uekitani, Kanako Yoshida, Megumi Harano, Isao Sakaida

**Affiliations:** Department of Gastroenterology and Hepatology, Graduate School of Medicine, Yamaguchi University, 1-1-1 Minami-Kogushi, Ube, Yamaguchi 755-8505, Japan

## Abstract

*Background/Purpose*. The difficulties of endoscopic retrograde cholangiopancreatography in patients with Billroth II gastrectomy have been reported. We evaluated the usefulness of an anterior oblique-viewing endoscope and a double-balloon enteroscope for endoscopic retrograde cholangiopancreatography in such patients. *Methods*. From January 2003 to December 2011, 65 patients with Billroth II gastrectomy were enrolled in this study. An anterior oblique-viewing endoscope was used for all patients. From February 2007, a double-balloon enteroscope was used for the failed cases. The success rate of procedures was compared with those in 20 patients with Billroth II gastrectomy using forward-viewing endoscope or side-viewing endoscope from March 1996 to July 2002 as historical controls. *Results*. In all patients in whom the papilla was reached (60/65), selective cannulation was achieved. The success rate of selective cannulation and accomplishment of planned procedures in the anterior oblique-viewing endoscope group were both significantly higher than that in the control group (100% versus 70.1%, 100 versus 58.8%, resp.). A double-balloon enteroscope was used in 2 patients, and the papilla could be reached and the planned procedures completed. *Conclusions*. An anterior oblique-viewing endoscope and double-balloon enteroscope appear to be useful in performing endoscopic retrograde cholangiopancreatography in patients with Billroth II gastrectomy.

## 1. Introduction

Endoscopic retrograde cholangiopancreatography (ERCP) is an essential procedure for the diagnosis and treatment of pancreaticobiliary disease. However, because of anatomical changes, there are some difficulties in performing ERCP and associated procedures in patients with Billroth II gastrectomy. Difficulties are experienced especially in afferent loop intubation, reaching the papilla, selective cannulation, and performing endoscopic sphincterotomy (EST).

 Since the double-balloon enteroscope (DBE) was introduced by Yamamoto et al. [1], several studies have been reported in which DBE was used to perform ERCP with Billroth II gastrectomy [2–5]. These reports revealed a good success rate for reaching the papilla and selective cannulation. Despite its usefulness, there are some disadvantages, such as the balloon preparation required for DBE system and the management of two balloon systems, when performing ERCP using DBE [6].

 We have carried out ERCP with an anterior oblique-viewing endoscope in patients with Billroth II gastrectomy. Some studies have already reported the utility of an anterior oblique-viewing endoscope in ERCP and associated procedures [7, 8], but the standard procedures have not yet been established. 

In the present study, our strategy is described, and our results in performing ERCP in Billroth II gastrectomy patients with an oblique-viewing endoscope and DBE are reviewed. 

## 2. Materials and Methods

 From January 2003 to December 2011, 65 patients with Billroth II gastrectomy (51 men, 14 women; mean age 74.8 years; age range 57–92 years) were enrolled in this study (anterior oblique-viewing endoscope, AOE group). These patients' characteristics are shown in Table 1. In all cases, the anterior oblique-viewing endoscope (GIF XK200; working length, 103 cm; channel diameter, 2.8 mm; Olympus Medical Systems, Tokyo, Japan) was tried first. Of the failed cases, from February 2007, 2 were retried using a DBE (EC450-BI5; working length, 152 cm; channel diameter, 2.8 mm; FUJIFILM, Tokyo, Japan).

 For selective cannulation, a standard cannula, a tapered cannula, or a cannula with a flexible tip (PR233Q, Olympus Medical Systems) was used. EST was carried out following placement of a biliary stent. A needle knife (KD-441Q or KD 451 M, Olympus Medical Systems) was used to cut open the common channel and the terminal bile duct along the stent. Since our experience of perforation using a needle knife, EST was performed using a rotatable papillotome (Autotome RX, Boston Scientific, Natric, MA). A high-frequency generator (ICC200, ERBE, Tübingen, Germany) was used for electrocautery, and a medium-sized excision was made in Endocut mode. Endoscopic papillary balloon dilatation (EPBD) was used only in patients who had a coagulation disturbance or cases with insufficient EST. For EPBD, a dilation balloon (MAXFORCE, Boston Scientific; balloon length, 3 cm; maximum balloon outer diameter, 8 mm) was used.

 The results were evaluated based on the following: (1) the success rate of reaching the papilla of Vater; (2) the success rate of selective cannulation of the bile duct or main pancreatic duct; (3) early complications. Post-ERCP pancreatitis was defined according to Cotton's criteria [9]. Hemorrhage was defined as bleeding requiring local injection of hemostatic agents or clipping at the time of procedure or a few days after.

 The success rate of procedures was compared with those in 20 patients with Billroth II gastrectomy who had undergone ERCP using forward-viewing endoscope or side-viewing endoscope from March 1996 to July 2002 as historical controls. Details of the historical controls are also shown in Table 1.

 For comparison of the patients' characteristics and the success rate of procedures between the AOE group and the historical control group, chi-square test or Fisher's exact test with Yates' correction was used. A *P* value of less than 0.05 was considered statistically significant.

## 3. Results

 Of the 65 patients with Billroth II gastrectomy, the papilla of Vater was successfully reached in 60 patients (Table 2). The cause of failure in 4 patients was acute angulation of the anastomotic site, and in 1 there was a long afferent loop through which it was difficult to pass. After introduction of DBE in our institution, ERCP was performed in 33 patients with Billroth II gastrectomy (33/65, 50.8%). In all but 2 patients, planned ERCP was carried out successfully using only anterior oblique-viewing endoscopy. In the remaining patients, DBE was needed to reach the papilla of Vater because of a long afferent loop and a Braun's anastomosis.

**Figure 1:**
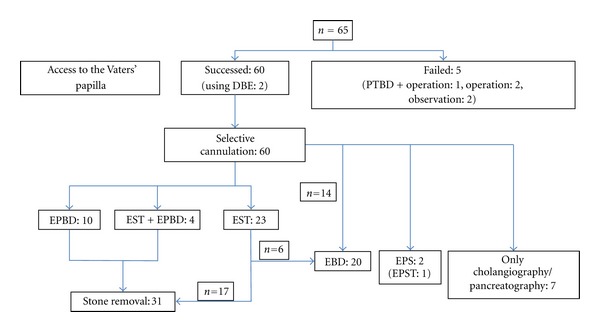
Summary of results. PTBD, percutaneous transhepatic biliary drainage; EBD, endoscopic biliary drainage; EPS, endoscopic pancreatic stenting; EPST, endoscopic papillary sphincterotomy.

 In all patients in whom the papilla was reached (60/65), selective cannulation was achieved (57 bile duct, 3 main pancreatic duct). EST was performed in 24 patients, EPBD was performed in 9, endoscopic pancreatic sphincterotomy (EPST) was performed in 1, and EPBD was performed after EST in 3 (Figure 1).

 Patient' characteristics did not differ significantly between 2 groups. In the AOB group, the success rate of reaching papilla did not differ significantly (Table 2). The success rate of selective cannulation and accomplishment of planned procedures in the AOB group were both significantly higher than that in the control group (100% versus 70.1%, 100 versus 58.8%, resp.). 

**Figure 2:**
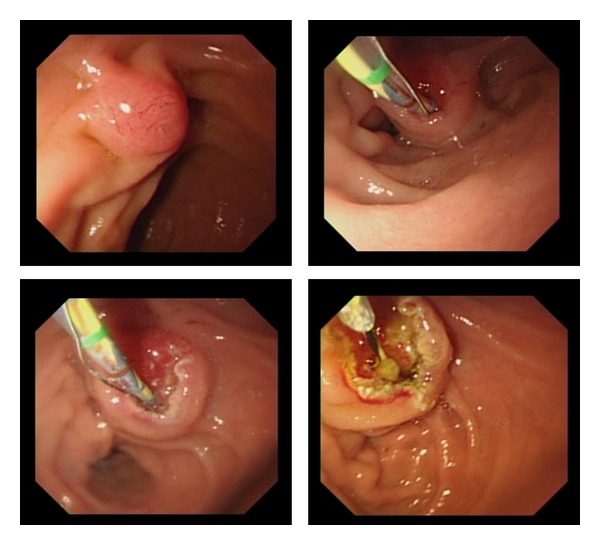
Sphincterotomy carried out with the “Autotome”, a rotatable papillotome. The blade was bent at the opposite side and directed towards the correct position.

 In the AOE group, six patients had early complications (Table 3). Postprocedure pancreatitis developed in 3 patients, and cholangitis developed in 2 patients, but the severity was mild. Perforation occurred in 1 patient after EST using a needle knife. Since then, a rotatable papillotome, “Autotome”, has been used for EST in our institution. We performed EST using the Autotome in only 1 patient. In that case, the Autotome could not be directed towards the correct position (5-o'clock position) by simply rotating the papillotome, so that a blade was needed to bend the opposite side. After bending the blade, EST could be performed safely with a good view (Figure 2).

## 4. Discussion

 Billroth II anastomosis is one of the reconstruction methods for distal gastrectomy. Because Billroth II anastomosis has a sufficient margin from the tumor and less morbidity, it is preferred for patients with lesions located higher in the stomach and high-risk patients [10, 11]. Patients with Billroth II gastrectomy appear to have a higher risk for gastric remnant cancer than patients with Billroth I reconstruction [12]; therefore, the number of Billroth II gastrectomies is decreasing in Japan. However, it is not rare to see patients with this type of anastomosis referred for ERCP because they have a higher incidence of biliary disorders, such as cholelithiasis.

In patients with Billroth II gastrectomy, ERCP and associated procedures are difficult because endoscopists encounter difficulties in afferent loop intubation, reaching the papilla, selective cannulation, and performing EST in a reverse direction. The type of endoscope is responsible for these difficulties. Although various types of endoscope, such as side viewing, forward viewing, and anterior oblique viewing, have been used in previous studies, the choice of endoscope for ERCP in patients with Billroth II gastrectomy has remained controversial.

The forward-viewing endoscope permits its operator to enter the afferent loop easily and safely because of the advantage to allowing him to see the lumen en face. But its shorter working length is a drawback in reaching the papilla, especially in patients with a long afferent loop and Braun anastomosis [4], and its lack of cannula elevator makes it difficult to cannulate the papilla. In contrast, the side-viewing endoscope has a long working length and cannula elevator. However, the fact that it is impossible to see the lumen en face makes it difficult to enter the afferent loop safely, and there are some reports of small bowel perforation associated with ERCP using a side-viewing endoscope [13, 14].

 The anterior oblique-viewing endoscope provides visibility as good as that of a forward-viewing endoscope. Moreover, its cannula elevator makes it easy to cannulate the desired duct selectively. In our study, the AOE group demonstrated a significantly higher success rate of selective cannulation and accomplishment of planned procedures as compared with the control group. These results also suggest the advantage of the presence of cannula elevator. Kikuyama et al. [7] and Nakahara et al. [8] have already reported the utility of an anterior oblique-viewing endoscope in ERCP for patients with Billroth II gastrectomy. They have also showed a good success rate of reaching the papilla (88.4–91.7%) and selective cannulation (94.7–100%) and a low complication rate. Unfortunately, in some patients in each study, the papilla of Vater could not be reached. 

 In recent years, ERCP using DBE in patients with a Billroth II gastrectomy has been reported [2–5], but its efficiency and safety have not yet been clarified. Furthermore, DBE requires specialized equipment and expertise that are not widely available. May et al. reported that it took 15.4 min to prepare the DBE system [6]. In contrast, the anterior oblique-viewing endoscope needs no special equipment to prepare.

 In this study, the papilla of Vater was reached in 92.3% of patients, but some patients in whom the papilla could not be reached remained. To improve the rate of reaching the papilla, DBE was used in 2 patients in whom the papilla could be reached using the anterior oblique-viewing endoscope. In both patients, the papilla was reached, and the planned procedures were completed. As described above, the anterior oblique-viewing endoscope has a cannula elevator, so we recently used it before using DBE.

From these results, the anterior oblique-viewing endoscope and the DBE seem to be very useful instruments for performing ERCP in patients with Billroth II gastrectomy. However, there is a common drawback to performing ERCP with the anterior oblique-viewing scope and DBE. It is the thin working channel (2.8 mm diameter), which limits the accessories available via the channel. Because each of these endoscopes has its own advantages and disadvantages, endoscopists must choose the endoscope based on an adequate understanding of the available endoscopes (Table 4).

 Several methods and instruments have been developed to enable EST in Billroth II patients [15–18]. Currently, the most accepted method consists of a needle knife sphincterotomy over a previously inserted endoprosthesis. We also have been performing EST using a needle knife, but we experienced minor perforation in one patient. In this case, one factor was unexpected deep cutting when the patient moved. After that case, we are trying EST using a rotatable papillotome. When the papillotome can be directed toward the correct position, the endoscopist may be able to perform EST safer and faster than when using the needle knife because a good view can be maintained.

 In conclusion, the anterior oblique-viewing endoscope is useful as an instrument for performing ERCP and associated procedures in patients with Billroth II gastrectomy. In addition, DBE can improve the rate of reaching the papilla in patients in whom the papilla cannot be reached using an anterior oblique-viewing endoscope. 

## Figures and Tables

**Table 1:** Patient's characteristics.

	AOE (*n* = 65)	control (*n* = 20)
Age (years)		
Mean	74.8	68.5
Range	57–92	49–91
Sex		
Male	51	14
Female	14	6
Diagnosis		
Choledocholithiasis	38	8
Obstructive jaundice	19	6
Pancreatic cancer	5	1
Gallbladder cancer	5	
Bile duct cancer	4	1
Hepatocellular carcinoma	2	
Carcinoma of papilla of Vater	1	3
Metastatic lymphadenopathy	2	
Malignant lymphoma		1
Bile leakage	2	
Pancreatic pseudocyst	1	
Chronic pancreatitis	2	2
Peribiliary cyst	1	
Others	2	4

**Table 2:** Success rate of procedures in Billroth-II gastrectomy patients.

	AOE		control		
	*n*	(%)	*n*	(%)	*P* value
Reaching the papilla of Vater	60/65	92.3	17/20	85.0	>0.05
Selective cannulation	60/60	100	12/17	70.1	<0.01
Accomplishment of planned procedures	60/60	100	10/17	58.8	<0.01

**Table 3:** Complications in the AOE group.

	*n*	(%)
Pancreatitis	3/65	4.6
Cholangitis	2/65	3.0
Hemorrhage	0/65	0
Perforation	1/65	1.5

**Table 4:** Comparison of endoscopes.

		Working length (mm)	Channel diameter (mm)	Visibility	Elevator
Side (posterior oblique)-viewing	JF260V, Olympus	1240	3.7	poor	+

Forward-viewing	GIF Q260, Olympus	1030	2.8	good	−
	DBE: EC450-BI5, FUJIFILM	1520	2.8	good	−
	SBE: SIF Q260, Olympus	2000	2.8	good	−

Anterior oblique-viewing	XK200, Olympus	1030	2.8	good	+
